# The Impact of Multidisciplinary In Situ Simulation-Based Team Training in the Operating Room on Patient Outcomes: A Scoping Review

**DOI:** 10.7759/cureus.87430

**Published:** 2025-07-07

**Authors:** Nada Sarwani, Saad Sulaiman, Dara O'Keeffe

**Affiliations:** 1 Anaesthesia, Salmaniya Medical Complex - Government Hospitals, Manama, BHR; 2 Internal Medicine, Mid Cheshire Hospitals NHS Foundation Trust, Crewe, GBR; 3 Surgical Affairs, RCSI (Royal College of Surgeons in Ireland) University of Medicine and Health Sciences, Dublin, IRL

**Keywords:** clinical outcomes, in-situ simulation training, multidisciplinary teams, operating room, patient outcome assessment, patient safety

## Abstract

A high-risk acute care area, such as the operating room (OR), requires seamless teamwork for the provision of safe care due to its fast-paced nature. Any breakdown in teamwork in this high-stakes setting could lead to adverse patient outcomes. Simulation-based team training strategies adopted from the principles of Crew Resource Management (CRM) from the aviation industry are a prominent modality used to train multispecialty teams in the OR. While sufficient evidence in the literature highlights the positive behavioral attitudes and improved non-technical skills of the OR teams, this review aims to examine and assess the impact of interprofessional point-of-care simulation training in the OR on patient safety and clinical outcomes.

A scoping review methodology was employed for comprehensive and iterative evidence research across five electronic databases. Methodological rigor was assessed using the Quality Assessment Tool for Studies with Diverse Designs, followed by thematic analysis of the extracted data and systematic mapping of the literature.

A total of 110 papers were screened, with 35 full-text articles assessed for eligibility. Twelve studies were included in the review with the identification of three recurring themes highlighting the impact of in situ simulation training in the OR on healthcare teams, clinical outcomes, and patient safety: 1) impact on team behavior, attitude, and non-technical skills; 2) identification of latent safety threats; and 3) clinical outcomes, morbidity, and mortality.

Simulation training in in situ settings offers multidisciplinary OR teams an opportunity to practice high-risk procedures in a controlled environment. It allows teams to overcome organizational barriers through the development of human factors and non-technical skills among healthcare professionals and identify latent safety threats to proactively address potential causes of error and harm, reflecting a constructive impact on patient safety. However, more research is warranted to demonstrate the direct influence of in situ simulation-based OR team training on clinical outcomes through standardized assessment tools, prompting initiatives aimed at improvement, and advocating for the incorporation of in situ simulation as a valuable component in the quality improvement cycle within the healthcare sector.

## Introduction and background

Simulation-based team training (SBTT) is an innovative educational tool that has been widely adopted across various industries, drawing principles from high-risk sectors such as aviation and utilizing concepts of Crew Resource Management (CRM) [1].

Gaining momentum in the past decade, training through simulation drills has been recognized as an effective educational tool to train both doctors and allied healthcare professionals, hence incorporating a multidisciplinary approach to enhance skills with encouraging and positive outcomes [2,3].

Simulation-based training is a pedagogical approach that reproduces real-life situations in a controlled environment, intending to enhance competency and skills[4]. It transcends conventional classroom instruction, allowing individuals to engage both physically and psychologically in managing emergencies and exercise decision-making capabilities within a replicated environment that closely simulates real-world circumstances. This pragmatic approach augments the retention of knowledge and enables individuals to acquire hands-on experience, make well-informed decisions, and refine their performance within controlled contexts [5].

Essential to the learning aspects of a simulation session is an expert-led, facilitated briefing and debriefing that helps to break down the common errors that one may encounter in a particular clinical context in question, and also trains the individuals in non-technical skills and team dynamics. This highlights that most errors and near-misses are largely attributable to a lack of appropriate communication, leadership skills, situational awareness, and proficiency in crisis management, rather than a lack of knowledge or clinical expertise [6].

On-site or point-of-care simulation involves training in the actual clinical settings, providing a high level of psychological immersion and accurate reflection of a real-life scenario, thereby impacting the participants' learning experience and degree of realism.

In situ simulation and healthcare teams

Healthcare organizations are well known to provide their services most prominently by formulating teams, and effectively functioning teams provide better outcomes in terms of patient safety, with higher rates of overall satisfaction [7]. Simulation training has been described in the literature as an effective tool for improving team performance and non-technical skills such as communication, coordination, and cooperation, and encouraging the promotion of a psychologically safe culture [8].

It is suggested that SBTT conducted in actual clinical environments increases participant buy-in and leads to improved teamwork both in terms of behavior and attitude during simulated events, and has a positive impact on team effectiveness [9].

There is also evidence of enhanced “human factor” or non-technical skills in experienced healthcare teams when CRM-based simulation training is provided in hospital settings as compared to training in a dedicated simulation lab [10].

In situ simulation in the operating room and patient safety

In the context of operating rooms (ORs), the pioneering work in 1994 by Gaba [11] on human factors and incidence of harm, particularly concerning anesthetists and the error-prone OR environment, is worth mentioning. Comparing the similarities of human behavior and attitudes and the level of hazard of the working environment between the pilots and the anesthetists, Gaba et al. describe a precept as “ hours of boredom, moments of terror.”

The role of human error and organizational failures leading to harm in the ORs is widely discussed in the literature [12]. The inherent similarities between the aviation industry and the OR environment as both being rapidly dynamic with high levels of uncertainty, multiple sources of information, reliance on indirect indicators with the frequent need to change plans and immediate consequences of actions, cognitive overload due to time and stress, all contribute toward increasing direct error and harm to the human population [12]. Additionally, the use of advanced technologies with multiple redundancies, intricate human-machine interfaces, and the coordination of numerous team members with varying priorities and hierarchical roles presents a complex challenge.

Findings from human factor literature highlight that in order to mitigate harm, it is beneficial to train individuals in their working environment, which helps to identify their system weaknesses and gaps in work processes [13-15].

Based on the core CRM principles of communication, leadership, situational awareness, and effective utilization of resources, in situ simulation education and training affords a distinctive opportunity for multidisciplinary and interprofessional teams to rehearse and enhance their collective skills in managing complex and high-stake scenarios, building confidence and increasing competency among the learners [16]. This is particularly relevant for identifying system lapses and failures in an acute care setting, such as the OR, and addressing safety concerns specific to the OR environment.

It is also a cost-effective strategy to allow the involvement of multispecialty teams and allied healthcare staff present in the OR with didactic learning opportunities during work hours without requiring dedication of extra time or space for training and educational purposes, and may also be beneficial for the junior trainees, students, and interns to increase their awareness and familiarity with the available resources and equipment.

However, while in situ SBTT has been extensively recognized across healthcare organizations as an invaluable educational tool with evidence of improved non-technical skills and positive reflections of immersive experience among the team members, particularly in acute care areas such as the OR [17,18], there is a need for further research correlating patient safety outcomes with this team training strategy.

The available literature does not provide clear conclusions regarding the effectiveness of multidisciplinary in situ simulation training in improving patient safety and clinical outcomes, as it is difficult to isolate its impact from that of other training modalities [19]. There is also a lack of robust data or studies that clearly demonstrate the correlation between in situ SBTT and the subsequent enhancement of patient safety measures and outcomes in critical care environments, hence emphasizing the need for further research in this area [20].

It is also equally important to understand the broad concept of safety, both from the provider and the receiver end of clinical care, as mismanagement of adverse events and harm can cause profound psychological trauma to patients, their families and caregivers, and the involved clinicians alike [21].

## Review

Research question

“What is the impact of multidisciplinary in situ simulation-based team training in the operating room on patient outcomes?” (PICO criteria)

We aim to conduct comprehensive research of the current literature, hypothesizing that SBTT of the OR personnel helps to yield better patient outcomes and improved clinical safety. It is also aimed at gaining a comprehensive insight into the current practices and identifying potential areas for further research and quality improvement in this important area of healthcare training, education, and patient safety.

Methods

Research Design and Description of Methods

Due to the wide, complex, and heterogeneous nature of the available evidence from the literature on in situ SBTT in the OR, a scoping review was aimed at exploring the proposed research question using the PRISMA-ScR methodology (PRISMA extension for Scoping Reviews) and the scoping review framework [22,23]. A comprehensive and iterative literature search was conducted, including all peer-reviewed articles and papers, followed by systematic data analysis, critical appraisal, synthesis of evidence from literature , and dissemination of results, identifying the gaps in current literature, and highlighting areas for further research.

Setting: Real-life (in situ) OR.

Participants: Multidisciplinary teams working in the OR, including at least one anesthesiologist and a surgeon (any level of training) and one nurse (scrub nurse, theater circulating nurse, anesthesia assistant nurse, or the recovery room (PACU) nurse). Gender or years of experience were not limiting factors for any team participant.

Data collection instruments, variables, & materials: For the purpose of data collection, the following key words and MeSH term sets were outlined according to the PICO criteria:

Population: multidisciplinary, interdisciplinary, health care teams, OR, operating theater

Intervention: simulation training, education, in situ, point-of-care

Outcome: patient outcome assessment, outcome assessment, health care, clinical outcomes, patient safety

Boolean operators AND/OR were applied to all search sets. 

To further refine our search and ensure the inclusion of all relevant studies, the following criteria were taken into consideration.

Inclusion criteria: All peer-reviewed articles and studies published in journals, books, or websites were included. However, the search was limited to the English language only, English being our language of instruction. All literature including the above-outlined setting and participants were considered eligible for the purpose of our research. Publications over the last two decades were included to ensure a wider and more comprehensive coverage of the research question, with the inclusion of studies in humans only. Team participants had to involve at least one member each from the nursing, anesthesia, and surgical disciplines. Studies with in situ or point-of-care simulation training components with reflection on patient outcomes were considered essential for the research process.

Exclusion criteria:* *Studies not meeting the inclusion criteria, such as papers published in a language other than English, were not included. The review also excluded any records flagged as ineligible by automation platforms, duplicate studies, and any other instances of duplication. Simulation training conducted in settings other than the OR was not considered eligible for the study. Also excluded were the studies where on-site simulation training was not associated with patient safety outcomes either directly or indirectly.

The study did not include gray literature and conference abstracts, as the research was aimed at evidence from peer-reviewed literature only.

Data collection procedures: As outlined in the scoping review framework [23], we aimed to research the primary domain of our subject in question via various resources such as the electronic databases, reference lists, and citation tracking, in order to ensure a wide and in-depth coverage of the literature on the same.

The electronic database search was aimed at reviewing citations from at least five databases, including CINAHL, Medline, Scopus, ERIC, and Web of Science.

Data storage and charting:* *As it was essential to keep track of all the databases searched and the resources utilized in the selection of all relevant studies, an Excel spreadsheet and EndNote were used to serve this purpose, dividing the data into subgroups such as author(s), year of publication, study location, type of study and methodology, setting, participants, intervention type or research tool, comparator (if any), outcomes measured or observed, and important results.

The standardized data extraction form provided by JBI (Joanna Briggs Institute) to minimize potential bias was utilized for this review. Each study was independently evaluated using the QATSDD quality assessment tool based on 16 methodological components [24,25].

Data Analysis, Collating, Summarizing, and Reporting the Results

Data were further classified according to the particular themes identified between different groups of charted studies during the analysis phase, and a descriptive-analytical approach was used to describe the findings and to provide a narrative overview of the results obtained.

Results

Search Results

**Figure 1:**
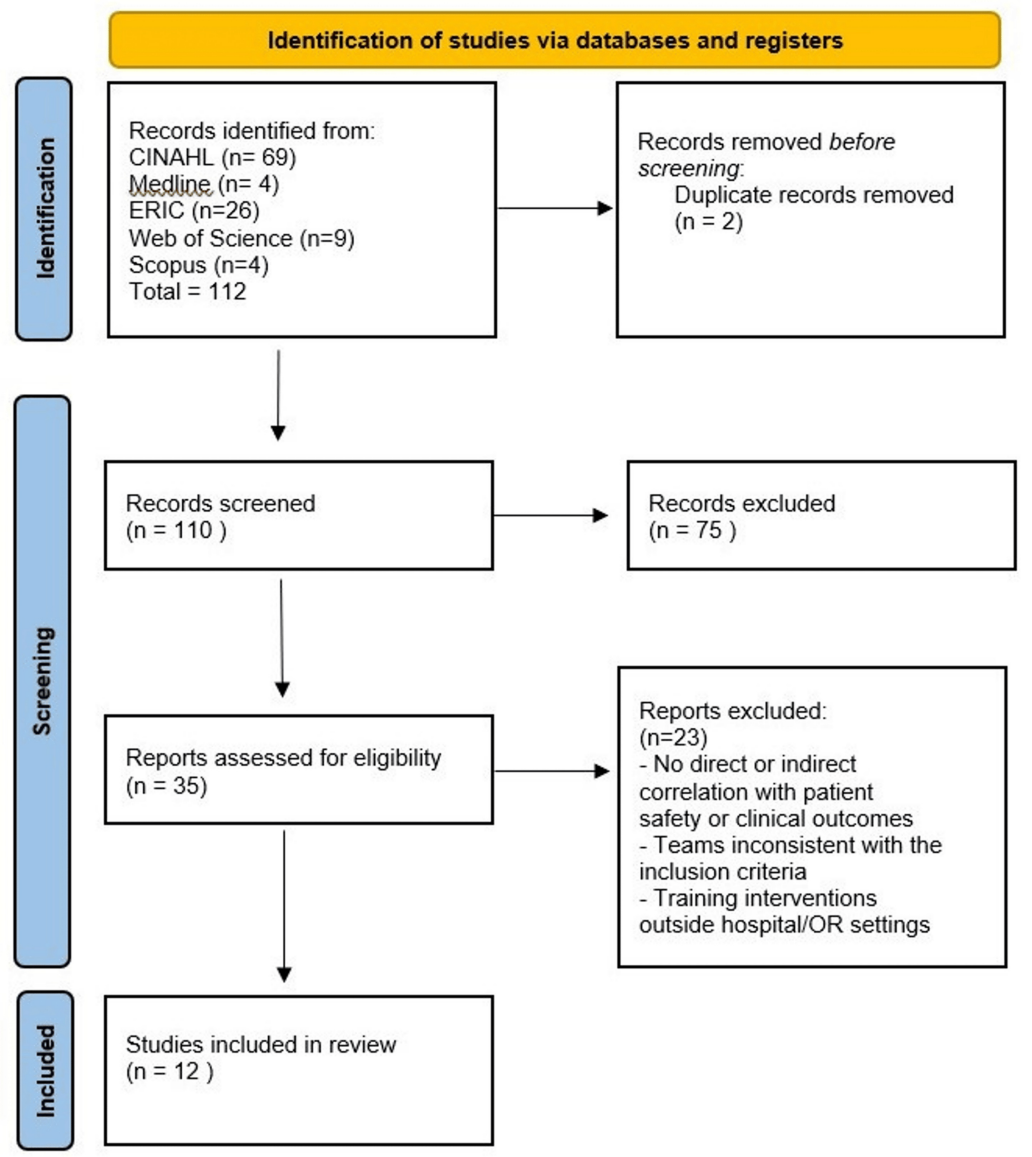
PRISMA Flowchart on Inclusion of Studies

The process of database search and evaluation of evidence for the purpose of research is shown in the PRISMA flowchart, depicted in Figure 1.

A total of 110 studies were screened for eligibility after a comprehensive search across five databases and the exclusion of duplicate records. Twelve studies were ultimately incorporated into the review, most of them having been published in the past decade (2014-2024), reflecting the culmination of the most recent evidence from the literature (66.66%, n=8).

The characteristic features of the studies reviewed are detailed in Table 1.

**Table 1:** Summary of the Studies Included for the Research. OR, operating room; ICU, intensive care unit; ED, emergency department; ODP, operating department practitioners; RN, registered nurse; NTS, non-technical skills; CRM, crew resource management; LSTs, latent safety threats.

Author/Year	Country	Type of Study	Setting/Participants	Intervention/Research Tool	Outcomes Measured/Observed
Mehta et al. (2006) [25]	USA	Prospective analysis	OR, ED/Vascular surgeons, ED physicians, anesthesiologists, operating room staff, radiology technicians	Simulation patients	Survival rate, morbidity, and mortality
Hamman et al. (2009)[26]	USA	Original research	OR, ED, ICU/physician, surgeon, anesthesiologist, nursing staff, pharmacist, respiratory therapist, and blood bank and lab technicians	CRM-based in situ simulation training	Team functioning, leadership effectiveness, and patient safety
Miller et al. (2009) [27]	USA	Observational study	Labour room, OR/obstetricians, labor, delivery, and special care nurses, neonatal nurse practitioners, anesthesiologists, certified nurse anesthetists, unit secretaries, and operating room staff	In situ simulation training	Behavioral aspects and NTS of nursing staff during crisis
Merién et al. (2010) [28]	Netherlands	Systematic review	Labor ward, delivery units, OR/midwives,obstetricians, anesthesia physicians, and trained nursing assistants	High-fidelity in situ simulation training	Clinical outcome (perinatal outcome, 5-minute Apgar score, hypoxic ischemic encephalopathy, maternal outcome), team knowledge, and performance
Fung et al. (2015) [9]	Canada	Systematic review	OR, ICU, ED, labor units/physicians, surgeons, nurses, emergency response team personnel, respiratory therapists, paramedics, and midwives	CRM-based simulation education, in situ high-fidelity simulation training	Interprofessional and interdisciplinary team effectiveness, behavior, attitude, and NTS, mean-weighted adverse outcome scores
Gillespie et al. (2017)[29]	Australia	Mixed-methods study	OR/surgical team members, including nurses (scrub persons, RN circulators, anesthesia nurses), anesthesia care providers, and surgeons	Team training program TEAMANATOMY including simulations, structured observations, survey, and semi-structured interviews	NTS, teamwork processes, and perception of safety climate and culture
Long et al. (2022) [30]	New Zealand	Observational study	OR/anesthetists, surgeons, technicians, theater nurses, and postoperative care nurses	Surgical in situ simulations, post-course report	LST identification
Wu et al. (2022) [31]	USA	Mixed-methods study	OR/anesthesiologists, perioperative nurses, surgical technologists, and patient care technicians	In situ mock Code Blue, simulation-based team training, videotaping, and survey	NTS, technical skills performance, self-reported confidence level
Evans et al. (2022) [32]	UK	Systematic mapping review	In-hospital settings (ED, ICU, OR)/Multidisciplinary teams (surgeons, physicians, nurses)	In situ simulation-based training	TeamSTEPPS performance scores, task completion, patient safety, identification of LST
Escher et al. (2023) [33]	Sweden	Prospective mixed-methods intervention study	OR/resident surgeon, resident anesthetist, OR nurse, nurse anesthetist, and associate nurse	Simulation-based teamwork training	Self-efficacy, situational motivation, and identification of barriers and risks to patients
Hassan et al. (2024) [34]	USA	Observational study	Medical and surgical ICUs, patient floors, and transplant unit/physicians, nurses, respiratory therapists, representatives from critical care, anesthesiology, otolaryngology, pulmonology, and ED	In situ simulation training, debriefing and audiovisual recording, and knowledge quiz	Adverse events, human errors, and LSTs
Towning et al. (2024) [35]	UK	Mixed-methods study	OR/ODPs, nurses, surgeons, healthcare assistants, and anesthesiologists	High-fidelity simulation training/pre- and post-training questionnaire	Staff preparedness and confidence

Study Design

Of the included studies, the mixed-methods research design was identified to be employed primarily (33.33%, n=4), followed by observational studies (25%, n=3) and systematic reviews (25%, n=3). One study was reported to engage a prospective analytic approach, with one study being an original research design.

Healthcare Settings

With ORs being the primary setting for our research, there was some overlap in healthcare settings, as some papers also looked at ICUs, ED, labor and delivery units, wards, and transplant units in addition to the OR.

Participating Countries

Most of the studies included in the review were primarily from the USA (41.66%, n=5), followed by the UK (16.66%, n=2). The remaining studies were conducted in the Netherlands (8.33%, n=1), Canada (8.33%, n=1), Australia (8.33%, n=1), New Zealand (8.33%, n=1), and Sweden (8.33%, n=1).

Thematic analysis

The thematic analysis of the included papers helped to highlight some key factors related to healthcare teams, patient safety, and impact on clinical outcomes when simulation training was employed as an in situ educational and training tool. However, most papers were found to have outcomes overlapping between these various themes.

Impact on Team Behavior, Attitude, and Non-technical Skills

In situ simulation training helped improve interdisciplinary team collaboration, communication, and coordination in the OR by creating a realistic clinical environment crucial for participant buy-in and immersive experience, improving learning, teamwork skills, and competencies as a unit. Effective crisis management and anticipation of the consequences of critical decisions in real time were essential to ensure patient and team member safety. This involved providing timely and frequent rehearsals of emergency situations, followed by prompt feedback and debriefing on specific, essential team competencies and skills that were crucial to safe practice. This approach targeted teaching teamwork competencies and non-technical skills effectively to individuals at all stages of the professional hierarchy, contributing to improved staff preparedness and enhanced patient safety in the OR [9,25-28,30-35].

Identification of Latent Safety Threats

Recognizing and managing complications is crucial for success. This requires identifying gaps in system processes that may lead to failure and adverse events, in turn posing a risk to patients and increasing the potential of harm.

Latent safety threats (LSTs) in OR settings were identified through in situ simulation scenarios, where incidents of suboptimal care during emergencies were observed. Point-of-care team training also played a crucial role in evaluating system readiness and staff preparedness. Direct observations, expert review of audiovisual recordings, pre- and post-training questionnaires, interviews, and team debriefing were the most commonly used interventional tools for the assessment and identification of LSTs and promotion of safety culture [29-32,34,35].

Clinical Outcomes, Morbidity, and Mortality

Of the included studies for review, only 25% of the studies (n=3) demonstrated a direct correlation of in situ simulation training effectiveness with enhanced patient survival, and reduced incidence of morbidity and mortality [9,25,28]. In 50% of the studies (n=6), an in situ multidisciplinary approach to training and education, rehearsed procedures by following standardized protocols, and coordination among healthcare providers significantly improved the staff confidence level and non-technical skills, reflected by enhanced team effectiveness and safety attitude among professionals. These were indirectly translated into improved patient outcomes and clinical safety by enhanced patient experience and provider satisfaction, with a reduction in the number of reported sentinel incidents and adverse events [29,31-35].

Discussion

The OR functions as a clinical microsystem within a healthcare organization. It is a high-risk, fast-paced work environment that necessitates seamless teamwork for the provision of safe care. In this high-stakes setting, any breakdown in team function could lead to adverse patient outcomes. Therefore, it is crucial to have efficient and dependable coordination of team interactions to prevent chaos and ensure the smooth delivery of patient care in the OR. Without effective communication and coordination, the risk of clinical errors and complications increases, which can ultimately lead to negative patient outcomes [11,12,36].

The human factors engineering principles, mostly frequently adapted from the aviation industry, are applied in healthcare organizations to reduce errors in complex systems, particularly in high-risk acute care areas, such as the OR [1].

Interprofessional OR team training and education using simulation is effective in teaching teamwork competencies to OR personnel and shows promising outcomes either directly or indirectly in improving patient safety.

This scoping review has allowed us to elucidate the most recent evidence from the literature, interlinking point-of-care simulation training of multidisciplinary healthcare teams in a high-risk OR setting with patient outcomes and the development of safety culture. The key findings of this review suggest the following:

Non-technical skills such as teamwork, leadership, management, communication, coordination, professional attitude and behavior, situational awareness, and a shared mental model between different members of healthcare teams are crucial for effective workflow and safe patient care.

Point-of-care simulation training enables the development of these non-technical skills in a familiar workplace environment, ensuring consistency, boosting self-confidence among staff, and preparedness to manage emergencies.

Development of human factor skills and immersive educational experience through in situ simulation drills can be indirectly translated into improved clinical outcomes by demonstrating better team performance and reduced number of errors, especially during charged situations.

The direct impact of in situ simulation training on patient safety has been demonstrated by the identification of LSTs and gaps in system processes, reflected by improved patient survival rates and reduced incidence of morbidity and mortality.

Gaps in Literature

Despite the encouraging and positive outcomes from the studies included in this review, evidence correlating patient safety and clinical outcomes with in situ simulation training and education is limited, as assessments have been made mostly by observational methods, self-reported, or by informal and non-standardized scoring systems [9,25,28]. Although this review has highlighted support for using SBTT and education in the clinical setting to promote the safety culture, a consensus has not been reached regarding the most effective tools for evaluating safety [10].

Moreover, the reflected rates of adverse events, mortality, and morbidity run the risk of being under-reported, hence compromising results. The overall influence on patient safety and clinical outcomes may also be multifactorial, as studies have failed to demonstrate in situ simulation training as a solo interventional tool affecting safety, indicating areas for further research [19].

Strengths and Limitations

This scoping review has several strengths, which may be noted. A comprehensive and structured search approach was adopted across five databases, and the methodological quality of the studies included was evaluated systematically, employing a predefined set of criteria. The studies included reflected the most recent evidence from the past decade to inform clinical practice. Additionally, the credibility of the findings is enhanced by the alignment of data from different sources into consistent themes.

However, as this review focused only on peer-reviewed literature in the English language, this may have overlooked the evidence from papers in languages other than English. The findings might have also been affected by publication bias, as unpublished studies and gray literature were not included. Additionally, certain studies were found to be constrained by methodological limitations such as a small number of participants, inadequate intervention exposure, and short follow-up period [25,28,29,31,34].

It is also recognized that the significant heterogeneity in various aspects of the research studies posed challenges in synthesizing and interpreting results.

Future Directions

To further promote the use of in situ simulation training as an effective educational and safety tool, it is essential to conduct well-designed trials that show clear benefits in its favor. Although previous studies have provided insight into the role of on-site simulation team training in patient safety, future research should aim to take things further by demonstrating that detecting and rectifying LSTs can prevent real-life patient safety incidents and improve institutional morbidity and mortality rates. A standardized approach to recording and grading the severity of LSTs, which is directly comparable to the scoring systems used for patient safety events, would facilitate this process.

Currently, no studies have been identified that highlight a cost-benefit analysis and fiscal benefits of in situ simulation team training as a patient safety tool. In addition to demonstrating its effectiveness, showing the cost-effectiveness of this educational strategy, particularly when compared to other methods, would greatly support its adoption.

## Conclusions

The outcomes of this study highlight the usefulness and dependability of in situ simulation training as a constructive strategy for educating healthcare professionals in recognizing potential gaps in system processes and threats to patient safety, thereby preventing harm and incentivizing initiatives for local improvement and well-being. This research might serve as a precursor to a complete systematic review, which could enhance evidence-based clinical practice and encourage a safety culture within an organizational framework.
